# Using electronic clinical records to investigate service use and in-patient care of adults with intellectual disability and/or autism spectrum disorder

**DOI:** 10.1192/bjo.2021.732

**Published:** 2021-06-18

**Authors:** Jennifer Mutch, Rory Sheehan, Louise Marston, Nomi Werbeloff, David Osborn, Angela Hassiotis

**Affiliations:** 1 NHS Lothian Learning Disability Service; 2 University College London

## Abstract

**Aims:**

To describe characteristics of adults with intellectual disability (ID) and/or autism spectrum disorder (ASD) accessing care in one mental health Trust.

To explore factors associated with in-patient admission/risk of re-admission within 12 months of discharge.

**Background:**

There is concern that adults with intellectual disability and those with autism spectrum disorder are frequently admitted to mental health hospitals. The evidence from NHS datasets suggests that this remains a significant issue and is associated with personal, social and economic costs.

**Method:**

Adults (≥ 18 years) with ICD-10 diagnosis of “mental retardation” and/or autism who had accessed care in the Camden and Islington Foundation Trust were identified using the Clinical Record Interactive Search (CRIS). The identification process was validated through cross checking of free text in the electronic clinical notes. We compared demographic and clinical characteristics and service use, including length of admission, of 315 individuals with ASD and 339 with ID (with or without ASD). Logistic regression was used to explore factors associated with in-patient admission and re-admission within 12 months of discharge.

**Result:**

A greater proportion of adults with ID (with or without ASD) had a diagnosis of psychosis, substance misuse, or dementia whereas diagnosis of anxiety disorder was greater in those with ASD. Antipsychotics and other psychotropics were twice as likely to be prescribed for the ID ± group. Admission to psychiatric in-patient care was greater in those with ID ± ASD (adjusted OR 4.00, 95% confidence interval (CI) 2.41-6.63), men (aOR 2.28, 95%CI 1.39-3.75), younger adults (aOR 0.98, 95%CI 0.97-1.00), and in those with a diagnosis of schizophrenia spectrum disorder (aOR 5.08, 95%CI 3.00-8.61), affective disorder (aOR 2.23, 95%CI 1.29-3.83), personality disorder (aOR 1.94, 95%CI 1.02-3.68), and record of previous inpatient admission (aOR 2.18, 95%CI 1.17-4.05). Having ASD alone was associated with a greater risk of re-admission within one year of discharge, although this difference was not statistically significant (aOR 0.70, 95% CI 0.32-1.52). Comorbid diagnoses of affective disorder or personality disorder were the only significant associations with re-admission (aOR 3.11, 95%CI 1.34-7.23 and aOR 8.28, 95%CI 2.85-24.04, respectively).

**Conclusion:**

These findings provide the first longitudinal investigation into the acute care pathway for adults with ID and/or ASD in the NHS. Replication in other trusts is now needed to inform “at risk of admission” registers and guide targeted interventions to prevent admission.

